# Biomechanical analysis of cadaver rabbit Achilles tendons after full transection and suture: Comparison of U-Tang 4-strand with the cross-locked version of U-Tang 4-strand suture technique

**DOI:** 10.1016/j.heliyon.2024.e38215

**Published:** 2024-09-20

**Authors:** Johanna Buschmann, Kimberly Feiner, Esteban Ongini, Jess G. Snedeker, Pietro Giovanoli, Maurizio Calcagni

**Affiliations:** a Department of Plastic Surgery and Hand Surgery, University Hospital of Zürich, Rämistrasse 100, 8091, Zürich, Switzerland; b University Clinic Balgrist, Orthopaedic Biomechanics, Forchstrasse 340, 8008, Zurich, Switzerland

**Keywords:** Suture, Cadaver, Rabbit Achilles tendon, U-Tang 4-strand suture

## Abstract

**Introduction:**

Tendon injures rank as the second most common hand injuries, and unsatisfactory repair can significantly impact a patient's everyday life. Over the years, various suture techniques have been studied in the pursuit of finding the optimal repair method. The ideal tendon repair should achieve maximum strength, while being easy to perform and minimizing tissue trauma. This study aims to compare the mechanical properties of the cross-locked U-Tang 4-strand suture to its unmodified version, the U-Tang 4-strand suture, to assess which technique offers greater repair strength.

**Methods:**

Sixteen Achilles tendons from New Zealand White rabbits were randomly assigned to one of two suture technique groups; an original U-Tang 4-strand suture or a cross-locked U-Tang 4-strand suture, both performed using a 4-0 Supramid thread. The specimens were tested in uniaxial tension after a preconditioning phase. Cross-sectional area, load until failure, gap formation, stiffness, elastic modulus, and failure stress were determined.

**Results:**

The standard U-Tang 4-strand suture withstood a maximum force of 25.48 ± 6.06 N, while the cross-locked version endured 33.90 ± 6.09 N. This indicates that the modified version demonstrated significantly greater strength (p = 0.021). The elastic modulus of the cross-locked U-Tang 4-strand suture (0.02 ± 0.003 MPa) was significantly higher than that of the original version (0.01 ± 0.006 MPa) (p = 0.028). No significant differences were observed regarding the cross-sectional area, load at 2 mm gap formation, stiffness and failure stress.

**Conclusion:**

Employing the cross-locked U-Tang 4-strand suture results in a significantly greater maximum force and elastic modulus compared to the original U-Tang 4-strand suture, utilizing the same thread and number of strands and knots. Therefore, the cross-locking version provides an alternative for achieving more stable initial repair strength.

## Abbreviations

APL: Abductor pollicis longus
CSA: Cross-Sectional Area
EDC: Extensor digitorum communis
EDI: Extensor indicis
EDM: Extensor digiti minimi
FDP: Flexor digitorum profundus
FDS: Flexor digitorum superficialis
FPL: Flexor pollicis longus
Group A: Tendons sutured following the conventional U-Tang 4-stand suture technique
Group B: Tendons sutured following the cross-locked U-Tang 4-strand suture technique
PBS: Phosphate buffer solution

## 1 Introduction

For a long time, tendon injuries were not repaired, and it only became common in the 1850s, but was still doubted to be of benefit for the healing [1]. Subsequently, tendon grafts were the preferred form of treatment, while sutures became the primary treatment only in the 1960s [2,3]. Particularly in Zone II of the hand flexor tendons, referred to as ‘no man's land’, repair results were so unstable that tendon grafting was the preferred choice for that purpose [4].

The original assumption that tendon injuries must be treated as an emergency and repaired immediately has been disproved. Nowadays, primary tendon repair is performed within the first 72 h. A planned suture, performed by a specialized surgeon, has been shown to be more effective than emergency repair [5].

Over the last few decades, multiple techniques and configurations have been studied. They vary in number of core sutures, knot placement, suturing material, peripheral circumferential suture, and the use of locking or grasping stitches [2,3,5,6]. The first tendon suture was conducted in 1874, and the search for the perfect technique continues to this day [7,8]. Comparing studies investigating different suture techniques is challenging due to the lack of standardized examination methods [2]. There are no international guidelines regarding suture technique, and each hospital has its own preferred approach [8,9].

Based on various studies, the ideal suture combines the following aspects: (i) high tensile strength to enable early mobilization, (ii) the suture is easy to execute, (iii) secure knots, (iv) tendon ends lying smoothly on top of each other with minimal gapping, (v) maintained blood circulation, (vi) gliding should not be impaired, and (vii) tendon manipulation is held to a minimum [2,4,7,10]. According to Rawson et al., the most important factor is the initial repair strength (time zero repair strength) [2].

The number of strands is significant [3]. Studies indicate that 4- and 6-strand sutures withstand greater force than 2-strand sutures. While 6-strand sutures are more stable than 4-strand sutures, they are more traumatic to the tissue, resulting in comparable outcomes [11]. Jo and Calfee suggest, based on various studies, that a minimum of four strands is necessary in the core suture for an early successful rehabilitation [1]. Moreover, knot placement can have a major impact. Positioning it between the stumps has a negative outcome for the healing process, whereas a placement on the tendon surface can compromise gliding. According to Langer et al., sutures with less knots are to be preferred [7].

**Fig. 1:**
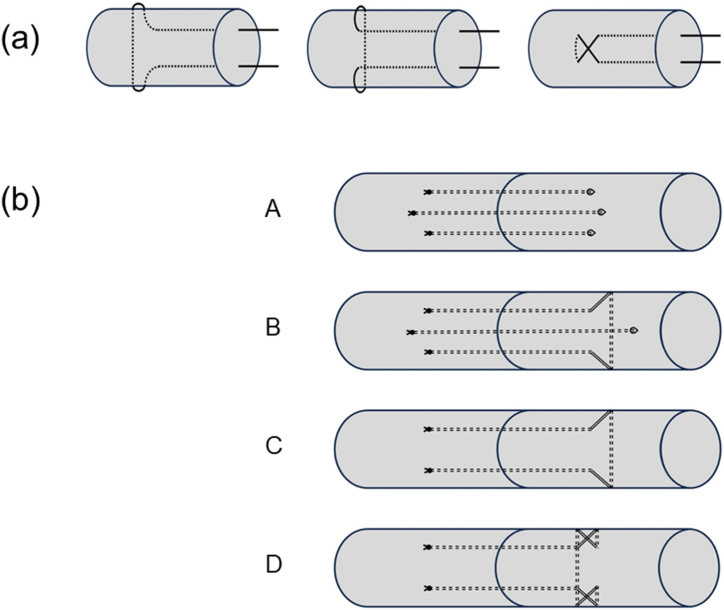
Illustration of different anchoring methods: grasping suture (left), locking suture (middle), cross-locking suture (right) **(a)**; and of the different Tang suture versions: (A) original Tang suture (1994), (B) M-Tang suture (2003), (C) U-Tang suture (2005), and (D) cross-locked U-Tang suture (2024) **(b)**.

Anchoring is beneficial to the suture strength [2]. Grasping or locking of the sutures has been shown to increase tensile strength by 5 N, tested on canine and human cadaver *flexor digitorum profundus* tendons [7,12,13]. Grasping sutures form a loop around tendon fibers, which is not a complete loop (Fig. 1 (a) left). On the other hand, locking sutures form a fully locked loop around the fibers (Fig. 1 (a) middle). When tension is applied, the locking suture tightens around the fibers, leading to a more stable anchor. Therefore, locking sutures are superior to grasping sutures in terms of repair strength but are more complicated to perform [2,6,7]. Various different locking configurations exist; one of them is the cross-locking method (Fig. 1 (a) right) [6].

In 1994, Tang et al. presented the 6-strand Tang suture, investigated for flexor tendon sutures in Zone II [14]. The method consists of three looped strands (Fig. 1 (b) A). In 1999, Tang et al. published a biomechanical comparison of the Tang suture with four different tendon sutures. The study showed that the Tang suture was able to withstand the highest ultimate strength and gap formation strength among the tested techniques [15].

In order to reduce the number of knots and sutures, Wang et al. reported a modified version of the Tang suture in 2003 (Fig. 1 (b) B): “the modified method consist of six longitudinal and two horizontal strands that from an ‘M’ configuration” [16].The modified version of the suture showed similar mechanical strength as the original suture technique [16].

In 2005, Cao and Tang reported another modified version of the Tang suture made by only one single looped suture forming a ‘U’ configuration (Fig. 1 (b) C). This modification makes the suture simpler to execute, and fewer knots lead to less adhesion. However, the four-strand suture is less stable than a six-strand version of the Tang suture [17].

In an effort to further improve the stability and efficacy of tendon repair techniques, this study investigates a new modification of the U-Tang 4-strand suture, where two cross-locked stitches are added (Fig. 1 (b) D).

Given that 25 % of tendon injuries still result in unsatisfactory outcomes, such as suture re-rupture or impaired gliding [2], and the current search for the perfect suture technique and configuration still continues [7], our study aims to provide insight into the mechanical properties of this modified suture technique, with a particular focus on reducing gap formation.

## 2 Materials and methods

### 2.1 Specimen

**Fig. 2:**
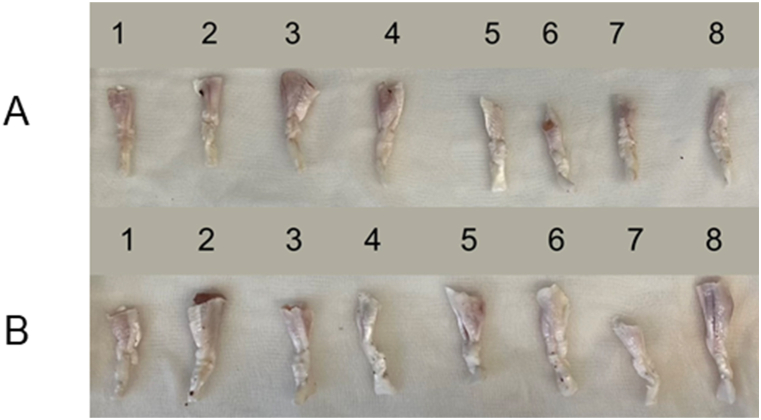
Tendons before testing, U-Tang 4-strand suture (A), cross-locked U-Tang 4-strand suture (B).

Achilles tendons from New Zealand White rabbits were used for this study, given that they exhibit similar mechanical strength as flexor tendons of the human hand and therefore present an adequate biomechanical model for the aim to compare the mechanics of two different suture techniques (Fig. 2) [18]. Specifically, these specimens had been collected from dead female New Zealand White rabbits that were included in a calvarial bone defect project. This corresponding project was licensed by the Animal Ethics Committee at local authorities in Switzerland (Canton Zurich ZH 115/2015 and 090/2021) [19,20] and had been preserved at −20 °C until further use. The tendons were thawed overnight and randomly assigned to two groups. Eight tendons underwent a conventional U-Tang 4-strand suture and 8 the cross-locked version of the U-Tang 4-strand suture method (Fig. 2). A 4-0 Supramid looped thread (50 cm, DRT 18, 3/8 taper cutting, 18 mm; RESORBA, Switzerland) was used for suturing. The required number of 8 animals per group was calculated with alpha = 0.05, power = 0.8 and on the basis of previous biomechanical analyses in the rabbit model [21], where effect size was approximately 1.4 for the biomechanical readouts.

After completing the sutures, the tendons were frozen again (−20 °C) until tensile testing. The tendons were thawed overnight at 4 °C. After 2 h at room temperature (21 °C), the tensile testing was performed.

### 2.2 Suture techniques

In this study the U-Tang 4-strand suture was compared to a cross-locked version of the U-Tang 4-strand suture. The original suture was conducted as described by Y. Cao and J.B. Tang:

“A looped locking suture is first anchored on the lateral aspect of the proximal tendon stump. The suture is then passed longitudinally inside the tendon and emerges on the lateral aspect of the distal tendon. The looped line is then passed transversely through the tendon and emerges on its contralateral lateral aspect. The suture is inserted into the tendon again and passed longitudinally back toward the proximal tendon. A knot is tied over the proximal tendon surface” [17].

**Fig. 3:**
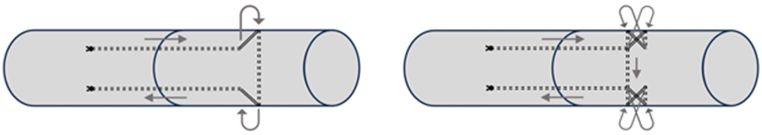
Illustration of the creation of the original U-Tang 4-strand suture (left) and the cross-locked U-Tang 4-strand suture (right).

The cross-locked version used in this study follows almost the same procedure. However, after the strand emerges in the distal part, before it is passed transversely, the suture gets cross-locked on the lateral aspect of the tendon. After the suture is passed transversely trough the tendon, another cross-locking stitch is performed (Fig. 3).

### 2.3 Before tensile testing

The cross-sectional area (CSA) of each specimen was determined before preparing the tendons for testing. It was measured by passing the specimen through a laser scanner (Sensor Head: Keyence, LK-G82; Display panel: Keyence, LK-GD500) three times each, using an in-house Protocol in MATLAB R2019a. After defining the CSA of each specimen, the tendons were prepared further for the biomechanical testing.

To prevent the specimens from slipping out of the clamps of the testing machine, they were wrapped in pieces of cloth, cut into 5–6 cm long and 1 cm broad strips, depending on the specimen. Each cloth strip was folded once in the middle, and then a drop of adhesive (Loctite 454, Henkel Corp., USA) was applied to each side of the strip, which was folded once more around the tendon.

After attaching the cloth strip to the ends of the specimen, the tendon was secured in the serrated clamps of the testing machine for measurements. To guarantee that they remained humid, the specimen were sprayed regularly using a phosphate buffer solution (PBS) throughout the testing process.

### 2.4 Tensile testing

The tendons were tested in uniaxial tension following the preparation process. The specimen were secured using serrated clamps on the testing machine (Zwick Z010, 1 kN load-cell, testXpert III (version V1.6); Zwick/Roell, Ulm, Germany). The first specimen was preconditioned with 10 cycles up to 10 N at a rate of 5 mm/min. Subsequently, it was tested in uniaxial tension until failure at a rate of 1 mm/min with a preload of 5 N. However, due to gap formation during the preconditioning phase, the protocol had to be modified. The other 15 tendons were preconditioned with 10 cycles up to 3 N at a rate of 10 mm/min and tested in uniaxial tension until failure at a 5 mm/min rate with a preload of 1.5 N.

The measurement phase, excluding the preconditioning, was recorded to be analyzed afterwards. The camera used for this purpose was a Canon EOS M50, equipped with a Nikon EOS M adapter and a telecentric lens (0.28x, Edmund optics).

### 2.5 Analysis and statistics

The videos were analyzed using Image J (version 1.54f). Since video recording commenced at the beginning of the testing until failure, the timestamps were utilized to precisely determine the force in the protocol. Image J was used to determine the initial gap formation (mm) and the point at which the gap reached a length of 2 mm.

The load until failure (N) was determined as the maximum load that was measured and could be retrieved from the measurement protocol. Stiffness (N/mm), elastic modulus (MPa) and failure stress (MPa) were calculated based on the values in the measurement protocol using Excel (version 16.83).

SPSS Statistics (version 29.0.0.0) was used to evaluate the significance of the parameters between the two tendon groups. Normal distribution was checked using the Shapiro-Wilk test and the Kolmogorov-Smirnov test. These tests were chosen for their sensitivity in detecting deviations from normality in small sample sizes. The homogeneity of variances was determined using the Levene Test. If all three tests yielded p values > 0.05, the two groups were analyzed parametrically using an independent samples *t*-test. In cases where the data were not normally distributed or there was no homogeneity of variances the groups were analyzed non-parametrically using the Mann-Whitney *U* test.

Data are presented as mean ± standard deviation using GraphPad Prism 9 (version 9.5.1). Significance was considered if p < 0.05 (∗), p < 0.01 (∗∗) or p < 0.001 (∗∗∗).

## 3 Results

### 3.1 Tensile testing

During the uniaxial testing to failure, each tendon was closely observed. The reason for the weakening of the tendon consistently occurred at the suture site; the tendons did not tear anywhere else.

**Fig. 4:**
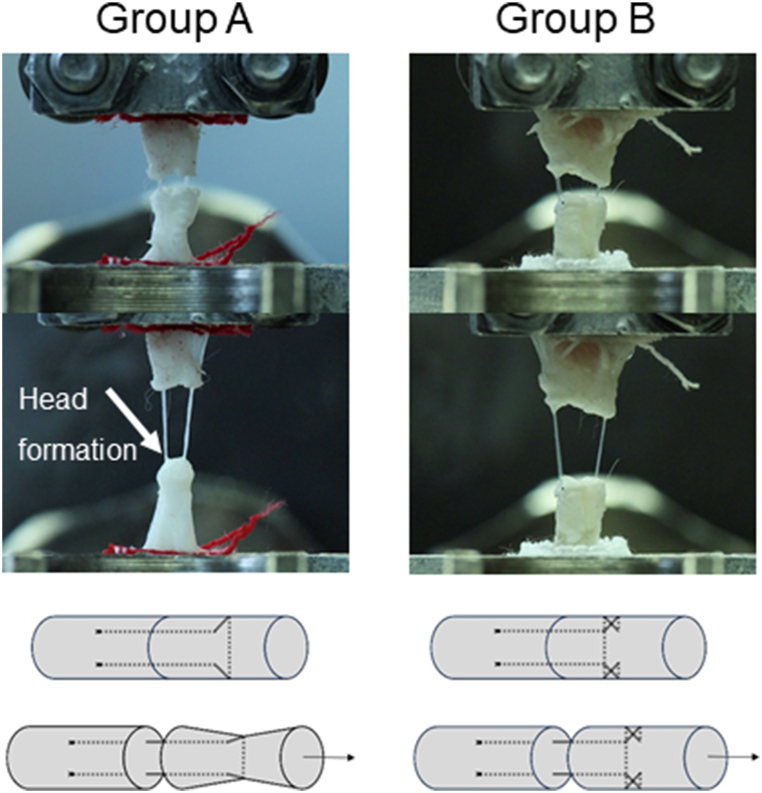
Head formation in one of the specimen of Group A (conventional original U-Tang 4-strand suture), no head formation in a specimen of Group B (cross-locked version). Below: Illustration of head formation in the original U-Tang 4-strand suture (left), illustration of no head formation in the cross-locked U-Tang 4-strand suture (right).

In Group A, which was sutured with the original U-Tang 4-strand suture technique, head formation was observed in every specimen. Head formation describes the tendon stump's rounding off caused by the tightening of the vertical suture part under tension (Fig. 4). In contrast, in the cross-locked U-Tang 4-strand sutured group (Group B), no head formation was observed. Furthermore, the reason for the suture failing to withstand more force was always either a knot tearing out or the thread itself tearing.

### 3.2 Biomechanical properties

**Fig. 5:**
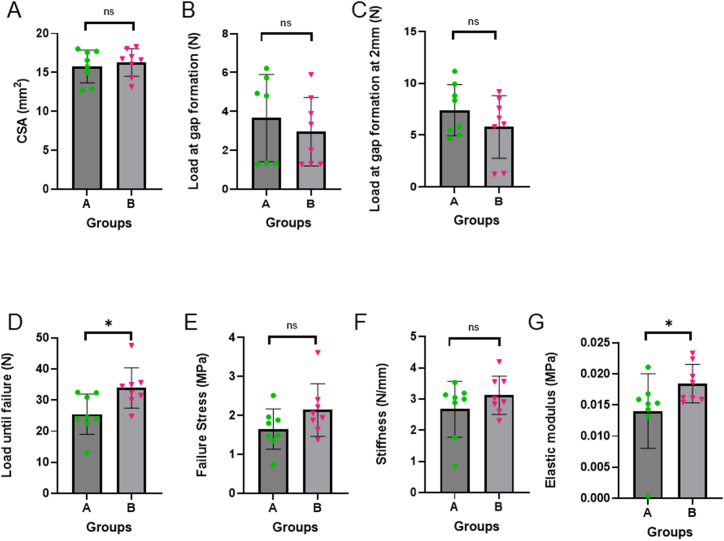
Properties of the repaired cadaver tendons. Cross-sectional are (CSA) **(A)**, Load until gap formation **(B)**, Load at 2 mm gap formation **(C)**, Failure load **(D)**, Failure stress **(E)**, Stiffness **(F)** and Elastic modulus **(G)**.

The CSA of Group A was 15.79 ± 1.98 mm^2^, and for Group B, it was 16.29 ± 1.65 mm^2^. There was no significant difference between the CSAs of the two groups (Fig. 5A). The forces causing the initial gap formation and a 2 mm gap formation, respectively, are shown in Fig. 5B and C. In Group A the initial gap formation of the first specimen was not documented because it occurred during the preconditioning phase. Therefore, only 7 out of 8 values were used for the analysis of gap formation in Group A.

Gap formation occurred at 3.66 ± 2.08 N in Group A and 2.96 ± 1.65 N in Group B (Fig. 5B). A lower force already resulted in gap formation in the specimen using a cross-locked U-Tang 4 strand suture. However, the differences between the two groups were non-significant.

A gap of 2 mm was formed at 7.40 ± 2.31 N in Group A and 5.78 ± 2.82 N in Group B (Fig. 5C). The cross-locked U-Tang suture formed a 2 mm gap at a lower force than the original U-Tang suture, but the values were not significantly different.

Ultimate force that the specimen could withstand before failure is shown in Fig. 5D. The ultimate force in Group A, using the original U-Tang suture, was 25.48 ± 6.06 N. The ultimate force in Group B (cross-locked U Tang) was 33.90 ± 6.09 N. The cross-locked U-Tang suture withstood a significantly greater force than the original suture technique (p = 0.021). The ultimate tensile stress of Group A was 1.66 ± 0.48 MPa. Group B measured a failure stress of 2.14 ± 0.63 MPa (Fig. 5E). Group B, therefore, withstood a greater stress level before failure. However, the difference was not significant.

Stiffness was calculated using the force-elongation curve. Group A (original suture) showed a stiffness of 2.86 ± 0.84 N/mm, and Group B a stiffness of 3.12 ± 0.57 N/mm, as shown in Fig. 5F. Group B demonstrated better resistance to deformation than Group A. However, the difference between the suture techniques did not prove to be significant. Finally, the elastic modulus was calculated from the stress-strain curve. The elastic modulus of the original U-Tang suture is 0.01 ± 0.006 MPa, while the elastic modulus of the cross-locked U-Tang suture is 0.02 ± 0.003 MPa (Fig. 5G). These values differ significantly (p = 0.028). Therefore, the cross-locked version is significantly more resistant to deformation than the original U-Tang suture.

## 4 Discussion

As the results of the biomechanical testing showed, the modified U-Tang 4-strand suture (Group B) withstood a significantly greater force to failure and had a significantly higher elastic modulus, but showed no benefit regarding the 2 mm gap formation.

Various studies outline the requirements for an optimal suture, one of them being tensile strength that allows early mobilization [2,4,7,10]. Studies have demonstrated that the force acting on tendons during passive movement is 2–4 N. Active movement against none to slight resistance causes a load of approximately 10 N. A powerful fist closure reaches 70–120 N. For active exercise treatment, the suture should withstand at least 19 N [5,7]. Consequently, the initial load until failure should be higher than that. The results of this study show that a cross-locked U-Tang 4-strand suture withstands an average of 33.90 ± 6.09 N. Therefore, the suture would allow early mobilization and withstand the corresponding loads.

Initial gap formation occurred at 3.66 ± 2.08 N in Group A and 2.96 ± 1.65 N in Group B, with a 2 mm gap at 7.40 ± 2.31 N in Group A and 5.78 ± 2.82 N in Group B. This indicates that both initial and 2 mm gap formation occur slightly earlier in the cross-locked version of the U-Tang 4-strand suture. However, the difference did not prove to be significant. When Yang et al. compared the mere Kessler suture with a locked modification of said technique, the force needed to form a 2 mm gap was higher in the locked version, using a fully locked loop as an anchor. However, they also found a non-significant difference as a result [22]. A possible explanation for the earlier occurrence in Group B of this study is that the cross-locking prevented the tightening of the transverse strand, preventing head formation but could have led to a high enough force immediately pulling the ends apart. In the work of Yang et al., on the other hand, the locked loop was able to tighten around the fibers as a first reaction to the tension, delaying the gap formation slightly. According to Yang et al.: “During tendon healing, a gap formation of >2 mm was associated with adhesion formation, decreased tendon gliding, and poor clinical results” [22]. This shows the importance of suture improvements that increase gap formation strength. However, the cross-locked U-Tang 4-strand suture does not positively influence the gap formation strength and in that aspect, does not represent a superior alternative to the original U-Tang 4-strand suture.

The elastic modulus of the original U-Tang 4-strand suture is 0.01 ± 0.006 MPa, whereas that of the cross-locked U-Tang suture is 0.02 ± 0.003 MPa. The significant difference between these values indicates that the modified version is more resistant to deformation. Tang et al. suggest that smaller deformation of the tendon enhances intratendinous vascular supply and synovial supply, which promotes healing. Additionally, the authors note decreased friction in tendons with a higher elastic modulus [23]. Therefore, the cross-locked U-Tang 4-strand suture is expected to facilitate better healing of the suture site and to enable smoother gliding compared to the original U-Tang 4-strand suture. These characteristics align with qualities identified in various studies as desirable for an ideal suture [2,4,7,10].

The modified version of the suture is an effective method to increase the ultimate force and resistance to deformation at the repair site, while keeping the number of strands and knots to a minimum. The downside is that the cross-locking stitch causes more material on the tendon surface, what could impair the gliding of the tendon [2].

Adding an anchor makes the suture more complicated to perform [6]. However, it also increases the stability, decreasing the risk of re-rupture. At the same time, it impairs gliding, increasing the risk of adhesion. This demonstrates the main problems of tendon repair: re-rupture and adhesion and how they limit each other [2].

The interpretability of the results is limited by the fact that the investigated specimen were cadaver tendons, and therefore, the physiological changes within the tissue after tendon lacerations did not take place, in other words, the rabbit Achilles tendons used in this study were native tendons from healthy rabbits. Although having very similar ultimate forces [24], which was a rationale to use them as a good mechanical model for flexors of the human hand, a further limitation is that we used tendons from rabbits and not from humans to assess the impact of the different repair techniques. Additionally, we had to go through two freeze-thawing cycles of the tendons that may adversely affect the tendon tissue to become brittle, although two cycles of freezing/thawing at only −20 °C and not −80 °C should not have affected the tendons too much, because it has been reported that even five freezing/thawing cylces at −80 °C did not impact the biomechanical strength, stress and stiffness of human *tibialis anterior*, *tibialis posterior*, *peroneus longus* and *medial* and *lateral* half of Achilles tendons [25,26]. If nevertheless unobservedly impacted by the freeze-thawing, the 16 specimen underwent the same procedure and are therefore comparable among each other. A further major obstacle was the lack of comparable studies. The number of examined techniques and configurations is huge, but they do not follow standardized protocols, making it very difficult to quantitatively compare the results to other studies. For further projects, the research would benefit from standardized protocols for hand tendon suture techniques performed on cadaver tendons. Finally, while the cross-locked U-Tang 4-strand suture increases tensile strength, its lack of improvement in gap formation strength indicates it may not be ideal for clinical situations where minimizing gap formation is essential for tendon healing. This limitation should be carefully considered alongside the benefits of increased tensile strength when deciding on the use of this technique.

Future studies should prioritize testing the cross-locked U-Tang suture in human tendons and in vivo models to evaluate its long-term effects on tendon healing and function. Additionally, investigating different suture materials and configurations could offer valuable insights for further optimizing tendon repair techniques.

## 5 Conclusion

In conclusion, while the cross-locked U-Tang 4-strand suture showed no significant effect on the gap formation, it proved to be an effective alternative to the original U-Tang 4-strand suture, increasing the elastic modulus and ultimate force of the repair site significantly in cadaver rabbit Achilles tendons. These biomechanical improvements represent potential benefits in future clinical scenarios, such as improved tendon healing, reduced re-rupture rates, and better functional recovery. Thus, a cross-locking step may be considered in orthopedic clinics to improve standard U-Tang 4-strand sutures.

## Funding

Not applicable.

## Data availability

Upon request, data are provided.

## CRediT authorship contribution statement

**Johanna Buschmann:** Writing – review & editing, Writing – original draft, Supervision, Project administration, Investigation, Data curation, Conceptualization. **Kimberly Feiner:** Writing – review & editing, Writing – original draft, Investigation, Formal analysis, Data curation. **Esteban Ongini:** Writing – review & editing, Supervision, Methodology, Investigation, Formal analysis, Data curation. **Jess G. Snedeker:** Writing – review & editing, Supervision. **Pietro Giovanoli:** Writing – review & editing, Supervision. **Maurizio Calcagni:** Writing – review & editing, Supervision, Methodology, Data curation, Conceptualization.

## Declaration of competing interest

The cadaver specimen were tendons from two previous studies, each of which had already undergone ethical review. They were collected from dead New Zealand White rabbits at the end-point. The veterinary application numbers were: ZH 115/2015 and ZH 090/2021. This corresponding project was licensed by the Animal Ethics Committee at local authorities in Switzerland (Canton Zurich ZH). Therefore, no additional veterinary application was required.

The authors declare the following financial interests/personal relationships which may be considered as potential competing interests:Johanna Buschmann reports financial support was provided by 10.13039/501100009396University Hospital Zurich. Johanna Buschmann reports a relationship with University Hospital Zurich that includes: employment. If there are other authors, they declare that they have no known competing financial interests or personal relationships that could have appeared to influence the work reported in this paper.
